# *Klebsiella pneumoniae* biofilm formation predicts its survival in human serum

**DOI:** 10.1128/mbio.00752-26

**Published:** 2026-06-03

**Authors:** Hadas Fulman-Levy, Liat A. Sinberger, Polina Geva, Jonathan Lellouche, Shiri Navon-Venezia

**Affiliations:** 1 The Department of Molecular Biology, The Faculty of Natural Sciences, Ariel University 42732, Ariel, Israel; 2 The Adelson School of Medicine, Ariel University 42732, Ariel, Israel; 3 Clinical Laboratories Department, Sanz Medical Center, Laniado Hospital 36902, Netanya, Israel; Universiteit Gent, Gent, Belgium

**Keywords:** *Klebsiella pneumoniae*, bloodstream infection, serum resistance, biofilm production, bacterial pathogenesis

## Abstract

*Klebsiella pneumoniae* is a prominent pathogen causing life-threatening bloodstream infections. Although biofilm formation and resistance to human serum are well-recognized virulence traits, their interrelatedness during *K. pneumoniae* bloodstream infections remains unclear. Here, we hypothesize that biofilm production is related to *K. pneumoniae*’s ability to thrive in human serum and, therefore, may predict the strains’ ability for serum survival. We analyzed 57 clinical, genetically diverse classical *K. pneumoniae* strains and characterized their survival and biofilm-producing ability in human serum. Serum survival patterns revealed three serum resistance categories—Low, Mid, and High. In addition, the biofilm biomass produced by the strains correlated with their serum resistance level (*P* < 0.001), and 3D biofilm visualization using confocal microscopy further confirmed that biofilm extracellular polysaccharide substances and biomass patterns were consistent with the serum resistance categories. Moreover, we revealed a direct correlation between the level of biofilm formation and the strain’s serum survival level (*R*^2^ = 0.696), a prerequisite for systemic *K. pneumoniae* dissemination. As biofilm formation in serum reflects both survival and biofilm-forming ability, we assessed biofilm formation in defined modified basal medium (BM2), to rule out serum-mediated killing, and discovered a strong and significant association between the serum resistance category and BM2 biofilm biomass (*P* < 0.0001). By applying regression models, we discovered that biofilm formation serves as a significant predictor for bacterial survival in serum. Overall, our findings establish biofilm production in *K. pneumoniae* as a biomarker of serum survival and may open a new avenue for predicting bloodstream infection risk in clinical settings.

**IMPORTANCE:**

Bloodstream infections caused by *Klebsiella pneumoniae* are devastating life-threatening infections worldwide. Understanding the survival strategies of *K. pneumoniae* in the bloodstream is critical for elucidating key aspects of bacterial pathogenicity and developing new diagnostic and therapeutic modalities. Although serum survival is a recognized virulence trait necessary to thrive in the bloodstream, the relationship between serum resistance and biofilm formation, a multicellular organization that may protect bacteria from bloodstream stressors, remains poorly understood. In this article, we demonstrate biofilm production in human serum by clinical classical *K. pneumoniae* strains for the first time and discovered a direct correlation between the level of biofilm biomass formation and the degree of serum survival in human serum and in defined modified basal medium. These findings offer insights into the importance of biofilm production in *K. pneumoniae* serum resistance and may be used to develop future therapeutic strategies targeting bloodstream infections.

## OBSERVATION

Bloodstream infections (BSIs) are a significant global health threat, with over two million deaths annually (1). To establish a BSI, a pathogen must withstand innate and adaptive immune mechanisms. One of the most important BSI pathogens is *Klebsiella pneumoniae* (Kpn), recognized by the World Health Organization as a global, life-threatening, multidrug-resistant pathogen (2). Kpn causes a variety of infections, including urinary tract infections (UTIs) (3) and BSIs associated with high mortality (4).

The ability to establish a BSI is reflected by the level of survival in serum, assessed *in vitro* by serum killing assay. Kpn serum survival varies widely among clinical isolates, influenced by various bacterial factors (5–7). Biofilm formation is a protective bacterial strategy to overcome environmental host stressors (8), and therefore, we hypothesize that biofilm formation promotes Kpn’s ability to thrive in serum.

While both biofilm production and serum resistance (SR) are well-recognized hallmarks for Kpn virulence (5, 9–11), most studies assess them in standard lab media (i.e., Luria broth), rather than under physiologically relevant conditions. Methodology differences in biofilm quantification could also underlie inconsistencies in Kpn biofilm-SR correlations (12–16), leading to ambiguity about the interrelationship between the two.

**Fig 1:**
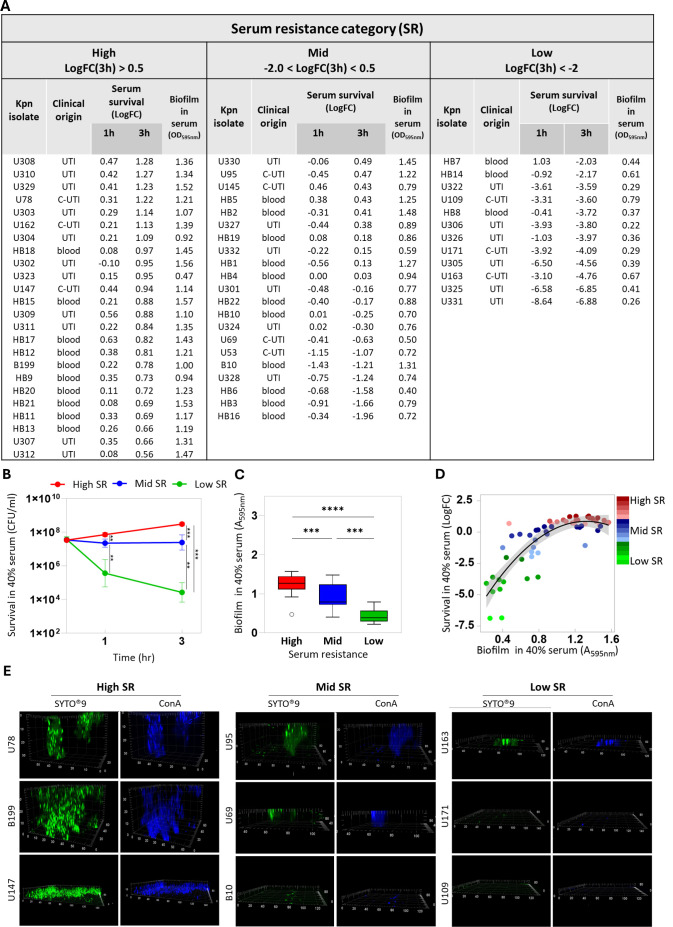
Serum resistance strongly correlates with *Klebsiella pneumoniae* (Kpn) biofilm formation in human serum. (**A**) Serum survival based on average log fold change (LogFC) from viable counts in 40% serum at 1 h and 3 h for each Kpn isolate. Pooled serum isolated from the collected blood was aliquoted and stored at −80°C. Aliquots were freshly thawed before each serum survival assay. The isolates were divided into three serum resistance (SR) categories: High, Mid, and Low based on the average LogFC at 3 h. The biofilm biomass was assessed using crystal violet staining after 24-h incubation in serum. (**B**) Three serum survival curves present the geometric mean ± SE (95% CI) of the strains classified in each SR category. Significance was determined using a two-way ANOVA with *post hocs* corrected for multiple comparisons. *P* ≤ 0.01 (**), *P* ≤ 0.001 (***), *P* ≤ 0.0001 (****). (**C**) Differences in biofilm biomass in serum presented in box plots, according to the three SR phenotype categories (High, Mid, and Low), evaluated by one-way ANOVA. *P* ≤ 0.01 (**), *P* ≤ 0.001 (***), *P* ≤ 0.0001 (****). (**D**) Scatterplots illustrating the non-linear relationship between bacterial serum survival (LogFC after 3 h in serum) and biofilm biomass formation (measured by crystal violet OD_595_) of the 57 Kpn strains. Each relationship was modeled using a second-order polynomial (quadratic) regression. The solid curve represents the fitted regression line, and the shaded area indicates the 95% CI. Within each category, the shading intensity is divided into five levels, corresponding to the bacterial serum survival from lightest (low LogFC) to darkest (high LogFC). (**E**) Confocal microscopy images of nine representative biofilms (three from each SR category) formed after 24 h in 40% serum. Biofilms were stained with Syto9 Live/Dead BacLight (green) and ConA Alexa Fluor 350 (blue) and imaged using a Zeiss LSM 700 confocal laser scanning microscope with a 63×/1.40 NA objective.

Here, we used 57 genetically diverse Kpn isolates from three clinical sources (community UTI, *n* = 10; hospital UTI, *n* = 23; and BSI, *n* = 24; Fig. 1A) to explore the relationship between serum survival and biofilm formation in human serum (see methods in Text S1). The genetic features of the isolates, including sequence type (ST), K-serotype, virulence score, and transferrable antibiotic resistance gene (ARG) content, were retrieved from whole genome sequencing (Text S1 and Table S1). Notably, we focused on classical Kpn strain STs for which biofilm-serum survival interactions are less studied compared with hypervirulent strains (17). We used 40% human serum in buffered saline gelatin (BSG; Text S1), based on survival curves performed in varying serum concentrations (results are not shown). Consistent with the established link between Kpn STs and virulence (18), our 57 genetically diverse clinical isolates exhibited marked phenotypic heterogeneity, characterized by a broad spectrum of SR and biofilm-forming capacities (Fig. 1A). Based on their serum survival levels, we grouped them into three distinct SR categories: High SR (log fold change [LogFC] > 0.5; *n* = 24), Mid SR (−2 < LogFC < 0.5; *n* = 21), and Low SR (LogFC < −2; *n* = 12), with all groups differing significantly (High SR vs Mid SR: *P* = 0.0003; High SR vs Low SR: *P* < 0.0001; Mid SR vs Low SR: *P* < 0.0001; Fig. 1B) based on 3-h LogFC values. The High SR group continuously proliferated in serum, reaching a mean survival of 1,000% at 3 h compared to the initial count (*P* < 0.0001), and the Mid SR group survived in serum with minor proliferation (mean survival of 177%; *P* = 0.32). The Low SR isolates’ survival, however, dropped drastically, to 1%, after 3 h (*P* < 0.0001) (Fig. 1B). We also observed that biofilm biomass was significantly different across SR categories (Fig. 1C; Table S2; *P* < 0.0001). Moreover, we discovered a strong and significant association between the SR category and biofilm biomass (Fisher’s exact test, *P* < 0.0001; Table S3). As the distribution of the data describing the SR and biofilm formation levels was non-linear, we applied a polynomial regression model. The predictor, biofilm formation in 40% serum, followed a second-order polynomial relationship with serum survival (Fig. 1D) and showed a significant quadratic association with survival (*Y* = −0.62 + 12.15*X* − 5.23*X*²; *P* < 0.0001 for both *X* and *X*², Fig. 1D; Table S4), accounting for 69.6% of the variance (adjusted *R*² = 0.696). The observed biofilm-serum survival relationship that we discovered in Kpn is consistent with observations in other major gram-negative BSI pathogens (19–22).

Three-dimensional visualization of the biofilms formed in serum, using confocal laser scanning microscopy followed by concanavalin A (ConA) and Syto9 Live/Dead staining (23) demonstrated that the biofilm architecture in serum strongly correlates with SR, supporting the physiological relevance of resistance categories (Fig. 1E). While serum-induced biofilm formation is documented in other pathogens (7, 24), our results demonstrate this phenotype for the first time in *K. pneumoniae*, suggesting that biofilm spatial organization may be a key driver of pathogenesis during BSI.

**Fig 2:**
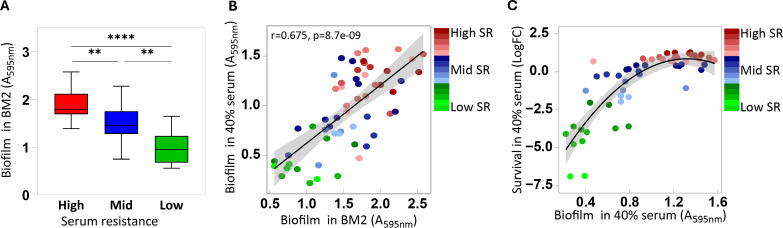
The relationship between biofilm formation and serum resistance (SR) using the defined modified basal medium (BM2). (**A**) The biofilm biomass produced by each of the 57 Kpn strains was assessed using crystal violet staining after 24-h incubation in BM2. Differences in biofilm biomass in BM2 presented in box plots according to the three SR phenotype categories (High, Mid, and Low) and compared by Kruskal-Wallis. *P* ≤ 0.01 (**), *P* ≤ 0.0001 (****). (**B**) Scatter plot showing the relationship between biofilm formation in serum and BM2 with a fitted linear regression model (*n* = 57, adjusted *R*^2^ = 0.495, *P* < 0.0001). (**C**) Scatterplots illustrating the non-linear relationship between bacterial serum survival (log fold change (LogFC) after 3 h in 40% serum) and biofilm biomass formation in BM2. Each relationship was modeled using a second-order polynomial (quadratic) regression. The solid curve represents the fitted regression line. The shaded area indicates the 95% CI. The dot colors represent the three SR categories (red: High; blue: Mid; green: Low). Within each category, the shading intensity is divided into five levels, corresponding to the bacterial serum survival from lightest (low LogFC) to darkest (high LogFC).

Because biofilm formation in 40% serum reflects both survival and biofilm-forming ability, we assessed biofilm formation in defined modified basal medium (BM2), containing 0.4% glucose, to control for serum-mediated killing (Text S1). We discovered that although average biofilm biomass in BM2 was approximately twofold higher than in serum (Fig. 2A; Table S2; analysis of variance (ANOVA); P< 0.0001), there was still a significant association between the initially assigned SR category and BM2 biofilm biomass (Fisher’s exact test, *P* < 0.0001 for each; Table S3). A linear regression model between biofilm in serum and in BM2 confirmed that biofilm biomass in BM2 predicted those in 40% serum (Fig. 2B, 95% confidence interval [CI]: 0.43−0.74, *P* < 0.0001, Table S4). This correlation indicates that biofilm-forming ability may contribute to SR independently of bacterial survival and that biofilm production is an intrinsic bacterial virulence trait, rather than a response to serum exposure. Biofilm formation in BM2 demonstrated a similar second-order polynomial relationship with serum survival (*Y* = −0.62 + 10.75*X* − 3.15*X*²; *P* < 0.0001 for *X*, *P* = 0.04 for *X*², Fig. 2C; Table S4), with the model explaining 48.8% of the SR variance (adjusted *R*² = 0.488).

Assessing biofilm in BM2—which is more practical and feasible than in human serum—could be easily implemented in clinical diagnostic laboratory settings using a simple *in vitro* assay to predict Kpn strain serum survival and associated BSI risk. Although we examined 57 diverse STs Kpn isolates from various clinical sources, similar to other studies (5, 13), we found no significant association between SR and isolate origin (Fisher’s exact test, *P* = 0.499, Fig. S1) and between capsule production (Kruskal-Wallis, *P* = 0.85; Fig. S2). Overall, the genomic data demonstrate high diversity of STs, K-serotypes, and transferable ARG content, independent of the SR category.

This proof-of-concept study demonstrates for the first time that Kpn can produce biofilm in human serum. Furthermore, the data provide the first evidence linking biofilm formation in defined media (BM2) and in human serum with serum survival. We discovered that biofilm formation accounts for a substantial proportion of the variance in serum survival (50%–70%), but beyond biofilm protection, additional factors may contribute to the strains’ abilities to thrive in serum. It should be noted that the *in vitro* serum survival assays used in this study do not fully represent the complex *in vivo* bloodstream environment, comprised of immune interactions, shear forces, and endothelial engagement. Future studies with larger data sets are warranted to fully explore classification-based predictive modeling.

### Conclusions

Together, our findings position biofilm formation as a key but not exclusive trait of Kpn persistence in the bloodstream and underscore the multifactorial nature of its SR. Using regression models, we identified biofilm-forming ability as a significant predictor of Kpn survival in human serum, linking this trait to the capacity of Kpn strains to persist in the bloodstream and establish BSIs. Overall, our findings suggest that high biofilm-producing capacity serves as a predictor biomarker for bacterial serum survival, highlighting the importance of monitoring these traits within infection control frameworks.

## SUPPLEMENTAL MATERIAL

Supplemental material mbio.00752-26-s0001.docx Text S1, Tables S1 to S4, and Fig. S1 and S2.

ASM does not own the copyrights to Supplemental Material that may be linked to, or accessed through, an article. The authors have granted ASM a non-exclusive, world-wide license to publish the Supplemental Material files. Please contact the corresponding author directly for reuse.
