# Supplementary material for: Klebsiella pneumoniae biofilm formation predicts its survival in human serum
Source: mBio. 2026 Jun 3;17(7):e00752-26. doi: 10.1128/mbio.00752-26 (PMC13344030; doi:10.1128/mbio.00752-26)
Supplement: Supplemental material — Text S1, Tables S1 to S4, and Fig. S1 and S2. [file mbio.00752-26-s0001.docx]

Supplemental material

**"*Klebsiella pneumoniae* biofilm formation predicts its survival in human serum”**

Hadas Fulman-Levy^1^, Liat A Sinberger^1^, Polina Geva^1^, Jonathan Lellouche^2,3^, Shiri Navon-Venezia^1,2^*

^1^The Department of Molecular Biology, The Faculty of Natural Sciences, Ariel University, Ariel, Israel ^2^The Adelson School of Medicine, Ariel University, Ariel, Israel ^3^Clinical Laboratories Department, Sanz Medical Center, Laniado Hospital, Netanya, Israel.

Corresponding author: *shirinv@ariel.ac.il

**Contents:**

**Text S1.** Materials and Methods.

**Table S1.** The clinical origin, sequence types and genetic features of the 57 *K. pneumoniae* study isolates.

**Table S2.** Statistical analysis of biofilm formation in correlation with the serum resistance categories.

**Table S3.** There is a significant association between serum resistance phenotype and bacterial survival outcomes.

**Table S4.** Regression model statistical values.

**Figure S1.** Biofilm formation and serum resistance (SR) levels were independent of clinical origin.

**Figure S2.** Serum resistance (SR) levels are independent of the isolates’ capsule production.

**Text S1. Materials and Methods**

**Bacterial strains and growth conditions**

*Klebsiella pneumoniae* (Kpn) isolates were identified using matrix-assisted laser desorption/ionization time-of-flight mass spectrometry (Vitek MS, Biomérieux, Marcy-l'Étoile). A total of 57 isolates were randomly chosen from different clinical sources to ensure representative sampling. Prior to further analyses, all frozen isolates were sub-cultured on Luria–Bertani (LB) agar plates and transferred to modified basal medium 2 (BM2) prepared as a 10×concentrate, which contained per liter of ultrapure water, 69.7 g K2HPO4 (Milipore), 29.9 g KH2PO4 (J.T. Backer), and 9.25 g (NH4)2SO4 (Sigma) and was adjusted to pH 7.0 and sterilized by filtration. Subsequently, a 10×glucose solution, containing 40.0 g/L D-glucose (Himedia), and a 50×MgSO4 solution, containing 50 mM MgSO4·7H2O (Merck), were prepared separately in DDW and sterilized by filtration. One liter of ready-to-use 1× BM2 was prepared by adding 100 mL of 10× BM2, 100 mL of 10× glucose, and 20 mL of 50× MgSO4 to 780 mL of DDW(1) containing ampicillin (100 μg/mL).

**Multilocus sequence typing using Nanopore WGS**

Genomic DNA from each strain was extracted using the QIAamp DNA Mini Kit (Qiagen). The genomic DNA was subjected to Oxford P2 (PromethION 2 Solo (P2 Solo), oxford Nanopore technologies, UK) sequencing (FLO-RPO114M) with Rapid Barcoding Kit 24 V14 (SQK-RBK114.24). Raw reads were processed in MinKNOW, including base-calling and trimming to remove adapter and barcode sequences. Quality filtering and error correction were performed with Filtlong (2), and draft genomes were assembled using Flye (3), and the quality of these assemblies was evaluated using  CheckM (4) and BUSCO (5) v6.0.0 which was run in genome mode against the enterobacterales_odb10 lineage database, which comprises of 440 conserved single-copy genes.

**Genomic characterization and sequence typing**

Assembled genomes were analyzed using Kleborate v3 (v3.2.4) (6) running the *Klebsiella pneumoniae* species complex preset (-p kpsc). Kaptive module was utilized to assign multilocus sequence types (MLST) and to predict capsule (K) serotype loci(7)

**Collection and preparation of human serum**

Human serum was collected from ten healthy adult volunteers using serum separation tubes containing a clot activator (Vacuette®, Greiner Bio-One). Blood samples were incubated (4°C for 1 h to allow clot formation) and subsequently centrifuged (1,500 rpm, 15 min). Pooled serum was carefully collected and stored at −80 °C until use.

**Human serum survival assays**

Survival curves in human serum were performed based on a previous method (8) with several modifications. Kpn strains were grown in BM2 to logarithmic phase (OD A_600_ - 0.6 to 0.8), washed with PBS, and resuspended in buffered saline gelatin (BSG) (0.85% NaCl, 0.03% KH_2_PO_4_, 0.06% Na_2_HPO_4_, and 0.01% gelatin) prior to the assay. The survival curves were performed in 96-multiwell plates with an initial bacterial concentration of 0.05 OD and incubated in 40% human serum in BSG at 37°C for 3h. The log fold change (LogFC) was calculated as Log_10_[(CFU/ml in serum) – (CFU/ml in BSG)] following viable counts performed at 1h and 3h. The serum killing assay for each Kpn strain was performed in at least three independent experiments.

**Quantitation of biofilm formation**

Biofilms produced in human serum or in BM2 were quantified using the crystal violet assay (9). A single colony of each Kpn strain was cultured in BM2, and after overnight growth, was adjusted to 0.05 OD in BM2 or washed three times with PBS and cultured in 40% serum (in BSG). Biofilm assessment was performed after incubation (37֩C, 24h) with 0.1% crystal violet (Sigma-Aldrich, USA) and quantification was performed using a plate reader (595nm, infinite M200PRO, Tecan Männedorf Switzerland). All biofilm experiments were performed in three biological replicates for each strain, with three technical replicates each. KpnB199 biofilm production (OD 595 nm) data was used to normalize biofilm production level between biological repeats.

**Biofilm visualization using confocal laser scanning microscopy (CLSM)**

Biofilms of nine Kpn strains were produced as described above in 40% human serum on glass-bottom dishes (Cellvis). Following a static incubation of 48h at 37°C, biofilms were stained with Syto9, the Live/Dead® BacLight bacterial viability kit (green) (ThermoFisher, USA), and Concanavalin A (ConA) Alexa Fluor® 350 conjugate 5mg/mL (blue) (ThermoFisher) and visualized by CLSM (63×/1.40 NA objective; LSM 700; Carl Zeiss, USA).

**Capsule quantitation** Cell-associated capsular polysaccharide quantification in BM2 media, was performed as previously described using 1% Zwittergent 3–14 and sulfuric acid hydrolysis (10). Uronic acid concentration (μg/ml/A_600_ nm) was assessed using absorbance readings at 520nm and was calculated relative to glucuronic acid standard curve.

**Statistical analysis**

Statistical analysis of serum survival was performed using GraphPad Prism version 10.4.1 (GraphPad, USA). All other statistical analyses and correlations were conducted using R 4.2.2 statistical framework (11, 12), and regression results were visualized using the ggplot2 package (version 4.2.3) (13). Specific analyses and significant differences are detailed in the figure legends.

**S1 References**

1. Haney EF, Trimble MJ, Cheng JT, Vallé Q, Hancock REW. 2018. Critical Assessment of Methods to Quantify Biofilm Growth and Evaluate Antibiofilm Activity of Host Defence Peptides. Biomolecules 8:29.

2. Wick R. 2026. rrwick/Filtlong. C++.

3. Kolmogorov M, Yuan J, Lin Y, Pevzner PA. 2019. Assembly of long, error-prone reads using repeat graphs. Nat Biotechnol 37:540–546.

4. Parks DH, Imelfort M, Skennerton CT, Hugenholtz P, Tyson GW. 2015. CheckM: assessing the quality of microbial genomes recovered from isolates, single cells, and metagenomes. Genome Res 25:1043–1055.

5. BUSCO: Assessing Genomic Data Quality and Beyond - Manni - 2021 - Current Protocols - Wiley Online Library. https://currentprotocols.onlinelibrary.wiley.com/doi/full/10.1002/cpz1.323. Retrieved 6 January 2026.

6. Lam MMC, Wick RR, Watts SC, Cerdeira LT, Wyres KL, Holt KE. 2021. A genomic surveillance framework and genotyping tool for Klebsiella pneumoniae and its related species complex. Nat Commun 12:4188.

7. Wyres KL, Wick RR, Gorrie C, Jenney A, Follador R, Thomson NR, Holt KE. 2016. Identification of Klebsiella capsule synthesis loci from whole genome data. Microb Genomics 2:e000102.

8. Sharma SK, Fatma T, Thukral SS. 1999. A simple and rapid serum bactericidal assay and its evaluation in clinical isolates of Klebsiella pneumoniae. J Microbiol Methods 39:45–48.

9. Christensen GD, Simpson WA, Younger JJ, Baddour LM, Barrett FF, Melton DM, Beachey EH. 1985. Adherence of coagulase-negative staphylococci to plastic tissue culture plates: a quantitative model for the adherence of staphylococci to medical devices. J Clin Microbiol 22:996–1006.

10. Khadka S, Ring BE, Pariseau DA, Mike LA. 2023. Characterization of Klebsiella pneumoniae Extracellular Polysaccharides. Curr Protoc 3:e937.

11. RStudio Team. RStudio: Integrated Development Environment for R. RStudio, PBC (2021).

12. R Core Team . R: A Language and Environment for Statistical Computing. R Foundation for Statistical Computing; Vienna, Austria: 2022.

13. Wickham, H. Ggplot2: Elegant Graphics for Data Analysis. (Springer-Verlag New York, 2016).

| ***Klebsiella pneumoniae* genetic features** | | | | | | | | | | | | |  | |  | |
| --- | --- | --- | --- | --- | --- | --- | --- | --- | --- | --- | --- | --- | --- | --- | --- | --- |
| High-SR | | | | | Mid-SR | | | | | Low-SR | | | | | | |
| Kpn # | ST | K  serotype | Vir score | ARGs | Kpn # | ST | K  serotype | Vir score | ARGs | Kpn # | ST | K  serotype | | Vir score | | ARGs |
| 308 | 104 | 31 | 1 | 0 | 330 | 37 | 14 | 1 | 10 | HB7 | 17 | 25 | | 0 | | 8 |
| 310 | 163 | 12 | 0 | 0 | U95 | 1412 | 107 | 0 | 11 | HB14 | 3057 | 30 | | 1 | | 11 |
| 329 | 661 | 140 | 0 | 0 | U145 | 873 | 52 | 0 | 12 | 322 | 353 | 110 | | 1 | | 11 |
| U78 | 1593 | 137 | 0 | 10 | HB5 | 111 | 63 | 1 | 4 | U109 | 29 | 113 | | 0 | | 10 |
| 303 | 20-1LV | 28 | 0 | 15 | HB2 | 15 | 48 | 1 | 11 | HB8 | 111 | 45 | | 1 | | 5 |
| U162 | 219 | 114 | 1 | 0 | 327 | 636 | 55 | 1 | 0 | 306 | 268 | 20 | | 1 | | 12 |
| 304 | 147 | 51 | 4 | 20 | HB19 | 2407 | 25 | 0 | 5 | 326 | 353 | 110 | | 1 | | 5 |
| HB18 | 29 | 2 | 1 | 11 | 332 | 307 | 102 | 0 | 12 | U171 | 1999-1LV | 38 | | 1 | | 1 |
| 302 | 5715 | 139 | 0 | 0 | HB1 | 13 | 3 | 1 | 10 | 305 | 323 | 21 | | 0 | | 3 |
| 323 | 35 | 22 | 1 | 7 | HB4 | 485 | 183 | 1 | 11 | U163 | 348 | 62 | | 0 | | 6 |
| U147 | 429 | 27 | 0 | 12 | 301 | 17 | 38 | 0 | 7 | 325 | 469 | 139 | | 0 | | 12 |
| HB15 | 395 | 2 | 1 | 10 | HB22 | 268 | 20 | 1 | 13 | 331 | 835 | 47 | | 0 | | 3 |
| 309 | 35 | 22 | 1 | 2 | HB10 | 753 | 3 | 0 | 13 |  |  |  | |  | |  |
| 311 | 17 | 102 | 0 | 11 | 324 | 36 | 102 | 0 | 0 |  |  |  | |  | |  |
| HB17 | 29 | 2 | 1 | 11 | U69 | 1412 | 107 | 0 | 11 |  |  |  | |  | |  |
| HB12 | 39 | 62 | 1 | 12 | U53 | 11 | 23 | 0 | 13 |  |  |  | |  | |  |
| B199 | 327 | 39 | 1 | 7 | B10 | 327 | 39 | 1 | 7 |  |  |  | |  | |  |
| HB9 | 1799 | 16 | 1 | 10 | 328 | 440 | 127 | 0 | 3 |  |  |  | |  | |  |
| HB20 | 469 | 139 | 0 | 13 | HB6 | 395 | 2 | 1 | 9 |  |  |  | |  | |  |
| HB21 | 307 | 102 | 0 | 12 | HB3 | 22 | 9 | 0 | 6 |  |  |  | |  | |  |
| HB11 | 551 | 10 | 0 | 19 | HB16 | 753 | 3 | 0 | 13 |  |  |  | |  | |  |
| HB13 | 985-1LV | 39 | 1 | 11 |  |  |  |  |  |  |  |  | |  | |  |
| 307 | 29-2LV | 10 | 0 | 2 |  |  |  |  |  |  |  |  | |  | |  |
| 312 | 36 | 27 | 0 | 0 |  |  |  |  |  |  |  |  | |  | |  |

**Table S1. The genetic features of the 57 Kpn study isolates.** Sequence types (ST) were determined in silico based on assembled genomes obtained from Nanopore long reads WGS (Material and Methods Text S1). Sequence type (ST), Capsule K serotype (K serotype), Virulence (Vir) score, antimicrobial resistance genes count (ARGs).


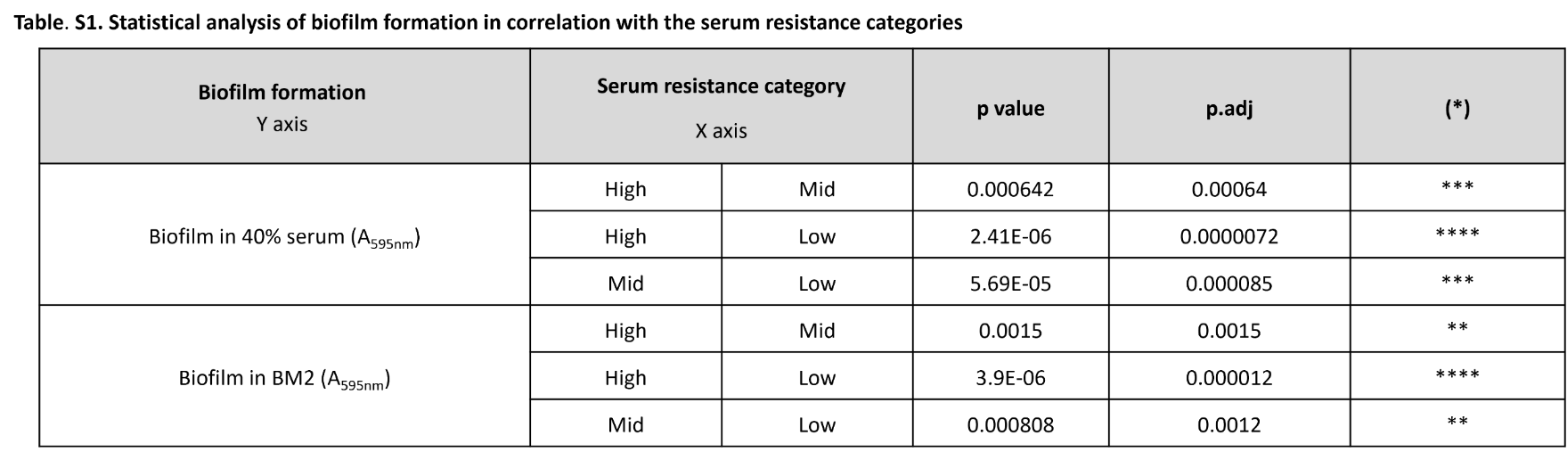


**Table S2. Statistical analysis of biofilm formation in correlation with serum resistance categories**. ANOVA tests were used to assess the differences between the serum resistance categories (High, Mid, Low) based on biofilm formation values in BM2. Kruskal–Wallis tests were used to assess the differences between resistance categories (High, Mid, Low) based on biofilm formation values in 40% serum, as the data were not normally distributed. ** p-value ≤ 0.01; *** p-value ≤ 0.001; **** p-value ≤ 0.0001.


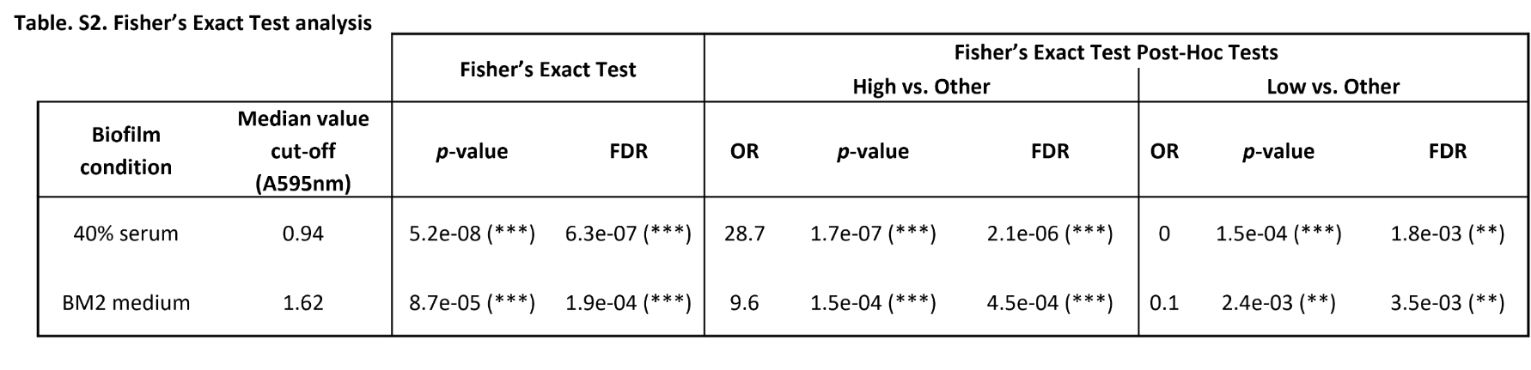


**Table S3. There is a significant association between serum resistance phenotype and bacterial survival outcomes.** Fisher's exact test was used to assess the association between serum resistance categories (High, Mid, Low) and bacterial survival outcomes. Survival (LogFC) was divided into high and low groups based on the median value. For each significant result from the initial Fisher's exact test, we conducted post-hoc Fisher's exact tests, comparing either the high resistance category against the combined mid and low categories, or the low resistance category against the combined high and mid categories. We calculated odds ratios (OR) and p-values for each comparison. To correct for multiple testing, we applied the false discovery rate (FDR) adjustment method; FDR < 0.05 was considered statistically significant. ** p-value ≤ 0.01; *** p-value ≤ 0.001.


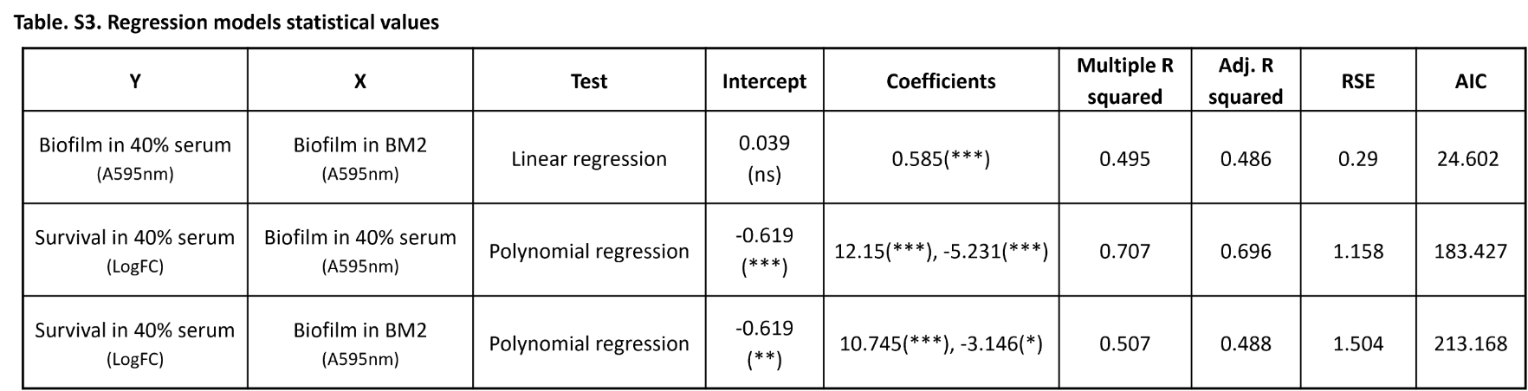


**Table S4. Regression model statistical values**. To investigate the relationship between biofilm formation and serum survival outcomes, we used three different univariate regression models. The first used linear regression with biofilm formation in 40% human serum as the outcome and biofilm formation in BM2 medium as the predictor. The second used bacterial survival (LogFC) as the outcome and biofilm formation in BM2 as a predictor, and the last used bacterial survival (LogFC) as the outcome and biofilm formation in 40% serum as a predictor. In each model, we examined both linear and polynomial (quadratic) regression models and used the best fitting one. The following evaluation metrics were used to select the best model: Akaike Information Criterion (AIC), adjusted R-squared (Adj. R-squared), and Residual Standard Error (RSE) (Table S2). ns: p-value > 0.05; *: p-value ≤ 0.05; **: p-value ≤ 0.01; ***: p-value ≤ 0.001.


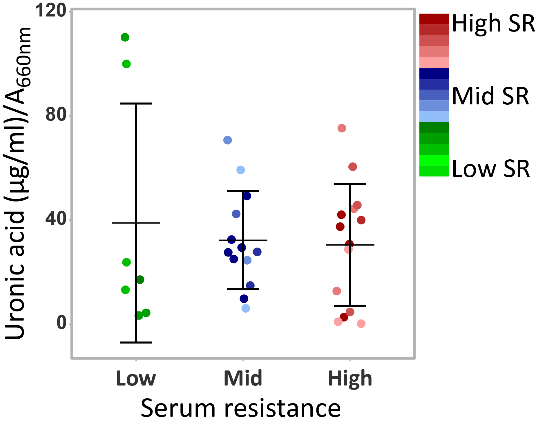

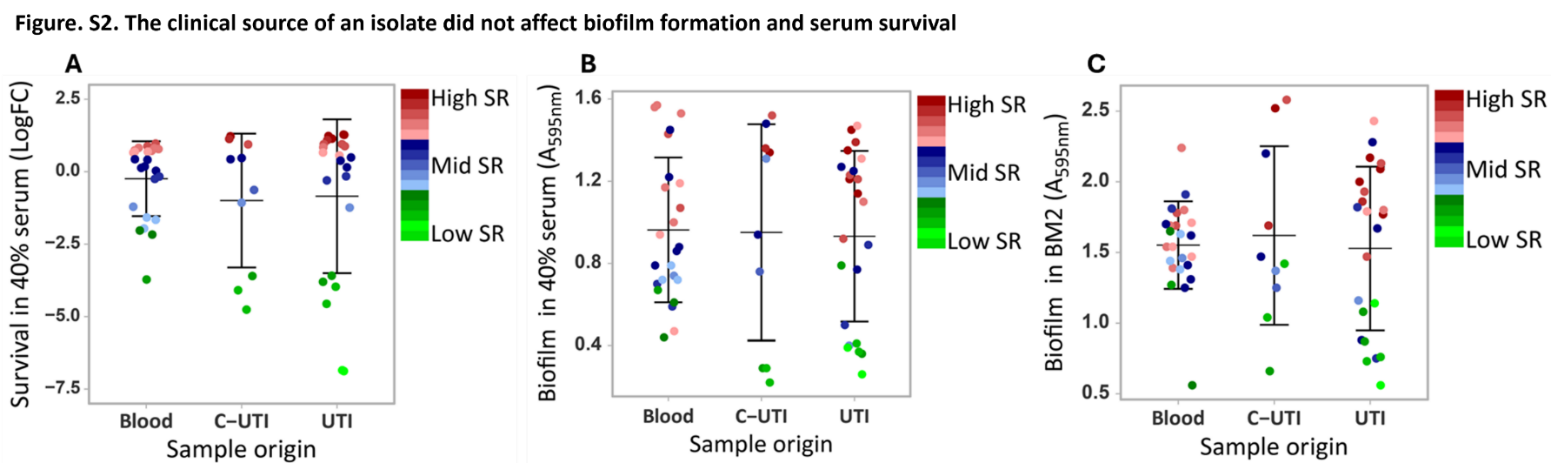


**Figure S2. serum resistance (SR) levels are independent of the isolates’ capsule production.** Capsule production of the strains distributed according to the strains’ sample serum resistance (SR) levels (High-SR, Mid-SR, Low-SR). The point colors represent the serum resistance (SR) category. Within each category, the shading intensity is divided into five levels, corresponding to bacterial SR (LogFC after 3h in 40% serum), from lightest (low LogFC) to darkest (high LogFC). The data is presented as the mean with whiskers representing the SD.

**Figure S1. Biofilm formation and serum resistance (SR) levels were independent of the isolates’ clinical origins.** (A) Survival of the strains (expressed as LogFC at 3 h); (B) Biofilm formation in 40% serum and (C) Biofilm formation in BM2 medium, distributed according to the strains’ sample origins (blood, community UTI (C-UTI) or hospital UTI (UTI)). The point colors represent the serum resistance (SR) category. Within each category, the shading intensity is divided into five levels, corresponding to bacterial SR (LogFC after 3h in 40% serum), from lightest (low LogFC) to darkest (high LogFC). The data is presented as the mean with whiskers representing the SD.
